# Coding Systems for Clinical Decision Support: Theoretical and Real-World Comparative Analysis

**DOI:** 10.2196/16094

**Published:** 2020-10-21

**Authors:** Nicolas Delvaux, Bert Vaes, Bert Aertgeerts, Stijn Van de Velde, Robert Vander Stichele, Peter Nyberg, Mieke Vermandere

**Affiliations:** 1 Department of Public Health and Primary Care Katholieke Universiteit Leuven Leuven Belgium; 2 Division for Health Services Norwegian Institute of Public Health Oslo Norway; 3 Department of Medical Informatics Ghent University Ghent Belgium; 4 Duodecim Publishing Company Ltd Helsinki Finland

**Keywords:** clinical decision support systems, clinical coding, medical informatics, electronic health records

## Abstract

**Background:**

Effective clinical decision support systems require accurate translation of practice recommendations into machine-readable artifacts; developing code sets that represent clinical concepts are an important step in this process. Many clinical coding systems are currently used in electronic health records, and it is unclear whether all of these systems are capable of efficiently representing the clinical concepts required in executing clinical decision support systems.

**Objective:**

The aim of this study was to evaluate which clinical coding systems are capable of efficiently representing clinical concepts that are necessary for translating artifacts into executable code for clinical decision support systems.

**Methods:**

Two methods were used to evaluate a set of clinical coding systems. In a theoretical approach, we extracted all the clinical concepts from 3 preventive care recommendations and constructed a series of code sets containing codes from a single clinical coding system. In a practical approach using data from a real-world setting, we studied the content of 1890 code sets used in an internationally available clinical decision support system and compared the usage of various clinical coding systems.

**Results:**

SNOMED CT and ICD-10 (International Classification of Diseases, Tenth Revision) proved to be the most accurate clinical coding systems for most concepts in our theoretical evaluation. In our practical evaluation, we found that International Classification of Diseases (Tenth Revision) was most often used to construct code sets. Some coding systems were very accurate in representing specific types of clinical concepts, for example, LOINC (Logical Observation Identifiers Names and Codes) for investigation results and ATC (Anatomical Therapeutic Chemical Classification) for drugs.

**Conclusions:**

No single coding system seems to fulfill all the needs for representing clinical concepts for clinical decision support systems. Comprehensiveness of the coding systems seems to be offset by complexity and forms a barrier to usability for code set construction. Clinical vocabularies mapped to multiple clinical coding systems could facilitate clinical code set construction.

## Introduction

Clinical decision support systems are considered to be an important vehicle for implementing new evidence and knowledge into daily practice [1,2]. Effective health care implies well-informed choices and decisions based on reliable evidence, with attention to individual needs and drawn from clinical experience [3]. Despite demonstrating rather small effects on adherence in clinical trials [4], clinical decision support systems are widely accepted as an important strategy for knowledge translation [5]. Clinical decision support systems generate patient-specific recommendations by matching individual patient characteristics to a knowledge base [6]; they are available in various formats and presentations, but an important variant of clinical decision support systems is guideline-driven, generating reminders based on formal rules and algorithms. The 3 essential components of a clinical decision support systems are (1) a knowledge base, (2) an inference or reasoning engine, and (3) an interface that can communicate with the user [7]. The knowledge base of a clinical decision support system consists of clinical practice recommendations that have been translated into machine-readable algorithms or artifacts. Artifacts are formal expressions of the recommendations in clinical guidelines. They include concepts from many different aspects from clinical practice, such as diagnoses, procedures, observations, or drugs. For the inference engine to be able to query the database of an electronic health record, each concept needs to be translated into a set of clinical codes also known as a clinical code set [8]. Collections of clinical codes, or clinical coding systems, are currently in use by electronic health records to represent clinical concepts, all with different finalities and purposes. Table 1 illustrates several of the current clinical coding systems used in electronic health records. Classifications include a form of taxonomy or structure of the included codes [8]. In some cases, this taxonomy is basic, such as those in the World Health Organization (WHO) family of classifications. For instance, in the International Classification of Diseases Tenth Revision (ICD-10), the structure is reflected by hierarchical alphanumeric codes. For instance, in ICD-10, the code for calculus of the kidney (N20.0) is a child of calculus of the kidney or ureter (N20) which is in turn a child of diseases of the genitourinary system (N). These types of taxonomies prevent clinical concepts from existing more than once in the classification but sometimes simplify more complicated concepts, such as pulmonary infections that could be classified as a pulmonary disease but also as an infectious disease. More complicated relationships are possible in ontologies, such as that in SNOMED CT, which includes not only “is a” hierarchy but also “has finding site” or “has causative agent [9].”

**Table 1:** Overview of some clinical terminologies used in electronic health records including their domain coverage and purpose. This list is not exhaustive.

Clinical coding system	Domain coverage	Type, purpose
SNOMED CT	Multiple areas (diagnoses, allergies, symptoms, etc)	Terminology, clinical documentation
International Classification of Diseases (ICD)	Diagnoses, some procedures	Classification, reporting
Current Procedural Terminology (CPT)	Procedures	Terminology, clinical documentation
Logical Observation Identifiers Names and Codes (LOINC)	Laboratory tests	Terminology, clinical documentation
Anatomical Therapeutic Chemical (ATC)	Drugs	Classification, reporting
International Classification of Primary Care (ICPC)	Diagnoses, reasons for encounter, some procedures	Classification, reporting

Most attention, when evaluating clinical decision support systems, is directed at ensuring technical interoperability and digitally structured data within the electronic health record as these determine the appropriateness of clinical decision support system alerts. Electronic health records currently use a variety of clinical coding systems to structure and represent clinical data, often with different purposes. Despite reports [8] on methods to translate clinical practice recommendations into interoperable artifacts and their code sets, it remains unclear whether currently used clinical coding systems are capable of representing the concepts needed for clinical decision support systems. Designers of terminology for clinical decision support systems have suggested that no one clinical coding system is capable of describing all necessary clinical concepts and that concurrent use of multiple terminologies is required [10]. The aim of this study was to evaluate whether currently used clinical coding systems are capable of efficiently representing the clinical concepts that are required for translating artifacts into executable code for clinical decision support systems.

## Methods

We used 2 separate methods—(1) theoretical and (2) practical evaluation using data from a real-world setting.

### Theoretical Evaluation

**Figure 1:**
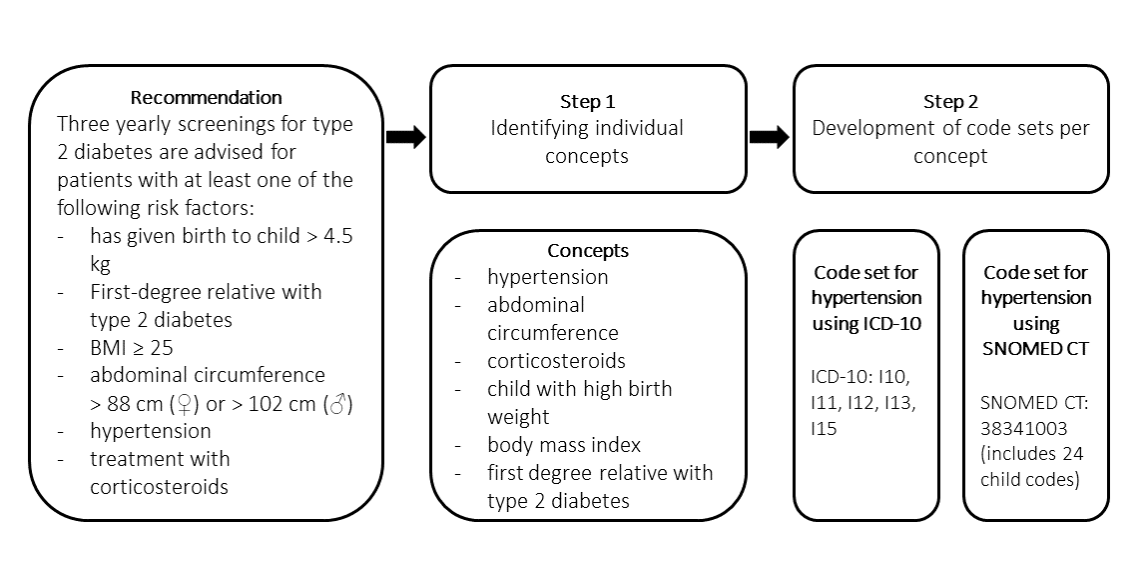
Process for the development of code sets for a guideline recommendation. BMI: body mass index; ICD-10: International Classification of Diseases, Tenth Revision.

We aimed to evaluate whether a selection of clinical coding systems was capable of representing the clinical concepts in a small set of recommendations and how many codes were required for this. First, we designed clinical decision support system artifacts based on 3 recommendations for preventive care which included concepts relevant to primary care [11]. We chose these recommendations because they were evidence-based and locally applicable, in addition, adherence to these recommendations was suboptimal. The recommendations used for this evaluation are described in Multimedia Appendix 1. We identified the clinical information described in the recommendations and isolated all individual clinical concepts to be used in the artifact. For each clinical concept, a clinical code set was constructed containing codes from a single system. We repeated this task for a selection of classifications, terminologies, and coding systems, including International Classification of Primary Care (ICPC)–2, International Classification of Diseases Tenth Revision (ICD-10) –Clinical Modification (-CM), SNOMED CT, Anatomical Therapeutic Chemical (ATC) Classification, and Logical Observation Identifiers Names and Codes (LOINC). An example of this process is illustrated in Figure 1.

However, not all coding systems were capable of representing the clinical concepts in an artifact. For example, for *acute exacerbation of chronic obstructive pulmonary disease*, there is no ICPC-2 code. The ICPC classification only includes a code for *chronic obstructive pulmonary disease* (R95) but does not allow specification of an acute exacerbation. The ICD-10 classification contains 2 codes for an acute exacerbation of chronic obstructive pulmonary disease: *chronic obstructive pulmonary disease with acute lower respiratory infection* (J44.0) and *chronic obstructive pulmonary disease with acute exacerbation, unspecified* (J44.1). Together, the codes J44.0 and J44.1 constitute the ICD-10 code set for *acute exacerbation of chronic obstructive pulmonary disease*. If the constructed code set was incapable of fully representing the clinical concept due insufficient granularity or overlap with other concepts, the code set was excluded from further evaluation. For SNOMED CT, we included all the codes, including child codes, that were required to fully represent the clinical concept.

### Practical Evaluation

Rather than constructing new code sets from recommendations, we studied the content of a large database of existing code sets used in an internationally available clinical decision support system, the Evidence-Based Medicine electronic Decision Support (EBMeDS, Duodecim Medical Publications Ltd). At the time of this study, EBMeDS contained 1890 concepts, and for each concept, a code set had been constructed using a large number of clinical coding systems currently in use. In addition to international coding systems, code sets also included local or national clinical coding systems and, in some cases, even electronic health record–specific proprietary coding systems. For each code set, we reviewed all included clinical coding systems and compared their usage. Clinical coding systems that were used in less than 3% of the code sets were not reported as these were always electronic health record proprietary coding systems.

## Results

### Theoretical Evaluation

For the 3 recommendations, we identified 21 different clinical concepts which we defined using 5 different clinical coding systems (see Multimedia Appendix 2 for the full code sets). Of these concepts, 10 were diagnoses, 3 were procedures, 2 were risk factors, 2 were laboratory tests, 2 were drugs, and 2 were vaccines. Table 2 show the distribution of the clinical coding systems for which we were able to create code sets describing the clinical concepts. In the case of the diagnosis concepts, for 9 out of 10 concepts, we were able to create a code set of SNOMED CT codes, for 8 out of 10 concepts we were able to create a code set of ICD-10 codes, and for 2 out of 10 concepts, we were able to create a code set of ICPC-2 codes. We did not find SNOMED CT codes for the concept *having given birth to a child over 4.5 kg*; we did not find ICD-10 codes for *presence of a cochlear implant* and *stress hyperglycemia*; and we did not find ICPC-2 codes for *asplenia*, *sickle cell disease*, *hemoglobinopathy*, *cerebrospinal fluid leak*, *presence of a cochlear implant*, *weak immunity*, *stress hyperglycemia*, and *having given birth to a child over 4.5 kg*. The SNOMED CT code sets included a median of 11 (range 1-219) codes, whereas the ICD-10 code sets included a median of 1 (range 1-3) codes.

**Table 2:** Code sets identified from 3 preventive care recommendations.

Concept type and coding system		Code sets per concept type^a^, n (%)	Codes per set, median (range^b^)
**Diagnosis (n=10)**			
	SNOMED CT^c^	9 (90)	11 (1-219)
	ICD-10(-CM)^d^	8 (80)	1 (1-3)
	ICPC-2^e^	2 2(0)	1.5 (1-2)
**Drugs (n=2)**			
	ATC^f^	1 (50)	1 (—^g^)
	SNOMED CT	2 (100)	108 (53-163)
**Vaccines (n=2)**			
	ATC	2 (100)	1 (—)
	SNOMED CT	2 (100)	3 (1-5)
**Risk factor (n=2)**			
	ICPC-2	2 (100)	1 (—)
	ICD-10(-CM)	2 (100)	1 (—)
	SNOMED CT	2 (100)	9 (9-9)
**Procedure (n=3)**			
	ICD-10(-CM)	2 (67)	1.5 (1-2)
	SNOMED CT	3 (100)	1 (1-37)
	LOINC^h^	3 (100)	5 (2-9)
**Investigation results (n=2)**			
	SNOMED CT	1 (50)	2 (—)
	LOINC	2 (100)	8 (7-9)

^a^The proportion of code sets per total number of concepts represents the proportion of clinical concepts for which a set of codes was found that matched the clinical concept.

^b^Minimum to maximum.

^c^SNOMED CT: Systematized Nomenclature of Medicine—Clinical Terms.

^d^ICD-10(-CM): International Classification of Diseases, Tenth Revision (–Clinical Modification).

^e^ICPC: International Classification of Primary Care.

^f^ATC: Anatomical Therapeutic Chemical Classification.

^g^Indicates that the range is defined by a single value.

^h^LOINC: Logical Observation Identifiers Names and Codes.

### Practical Evaluation

The majority of the predefined EBMeDS concepts were diagnosis or drug concepts, with very few risk factor or vaccine concepts. Table 3 shows the distribution of clinical coding systems used in the code sets for the clinical concepts including the number of individual codes.

**Table 3:** Code sets defined in the EBMeDS service.

Concept type and coding system			Code sets per concept type^a^, n (%)	Codes per set, median (range^b^)
**Diagnosis (n=790)**				
	**International**			
		ICD-10^c^	739 (93.5)	1 (1-53)
		ICPC-2^d^	223 (28.2)	1 (1-26)
		ICD-9-CM^e^	93 (11.8)	1 (1-550)
		SNOMED CT^f^	89 (11.3)	2 (1-36)
**Drugs (n=556)**				
	**International**			
		ATC^g^	481 (86.5)	1 (1-245)
		SNOMED CT	25 (4.5)	1 (1-2)
	**National**			
		Read codes (United Kingdom)	17 (3.1)	1 (1-5)
**Investigation results (n=317)**				
	**International**			
		LOINC^h^	116 (36.6)	1 (1-8)
		Nomenclature for Properties and Units	76 (24.0)	1 (1-9)
	**National**			
		KL Finnish classification for laboratory investigations	271 (85.5)	2 (1-60)
		Read codes (United Kingdom)	25 (7.9)	1 (1-4)
	**Proprietary EHR^i^**			
		Meldola Hospital measurement classification (Italy)	54 (17.0)	1 (1-2)
		SoSoeMe measurement classification (Belgium)	67 (21.1)	1 (1-3)
		Health One measurement classification (Belgium)	63 (19.9)	1 (1-3)
**Procedures (n=214)**				
	**International**			
		SNOMED CT	8 (3.7)	1 (1-43)
		ICD-9-CM	27 (12.6)	1 (1-16)
	**National**			
		Current Procedural Terminology	17 (7.9)	7 (1-42)
		Nordic procedure codes	179 (83.6)	1 (1-468)
	**Proprietary EHR**			
		Quantros Organization (United States)	7 (3.3)	1 (—^j^)
**Risk factors (n=2)**				
	**International**			
		ICD-10	1 (50.0)	1 (—)
	**Proprietary EHR**			
		Health One measurement classification (Belgium)	1 (50.0)	1 (—)
**Vaccines (n=11)**				
	**International**			
		ATC	11 (100.0)	1 (1-12)
	**National**			
		ROKVALM Finnish vaccination codes	9 (90.9)	7.5 (1-19)
		ROK Finnish vaccination codes	8 (81.8)	1 (1-7)

^a^The proportion of code sets per total number of concepts represents the proportion of clinical concepts for which a set of codes was found that matched the clinical concept.

^b^Minimum to maximum.

^c^ICD-10: International Classification of Diseases, Tenth Revision.

^d^ICPC: International Classification of Primary Care.

^e^ICD-9-CM: International Classification of Diseases, Ninth Revision–Clinical Modification.

^f^SNOMED CT: Systematized Nomenclature of Medicine—Clinical Terms.

^g^ATC: Anatomical Therapeutic Chemical Classification.

^h^LOINC: Logical Observation Identifiers Names and Codes.

^i^EHR: electronic health record.

^j^Indicates that the range is defined by a single value.

## Discussion

### Principal Findings

In the theoretical evaluation of clinical coding systems, we found that SNOMED CT and ICD-10 were capable of describing the majority of diagnosis concepts; however, for some clinical concepts, a very large number of codes was required. In our theoretical analysis, SNOMED CT was superior to ATC for drug concepts, which in our small set was entirely due to the fact that ATC does not always define a route of administration. The small sample of code sets for drug concepts probably exaggerated the superiority of SNOMED CT in comparison to ATC. The shortcomings of ATC—sometimes lacking a route of administration and not always including all substances of compound drugs—are probably less influential than our evaluation suggests. The procedure concepts in this study could all be mapped to SNOMED CT and LOINC. The only instances where SNOMED CT did not fully represent clinical concepts were investigation results. Code sets with SNOMED CT codes often included a lot more codes than those included for other coding systems, with a median of 108 codes for drug concepts. Constructing code sets with this number of codes may challenge the feasibility of this task. When compared to the results of the practical evaluation of current code sets in the EBMeDS database of clinical concepts, SNOMED CT was noticeably less present. Only 11.3% of diagnosis concepts (89/790) were mapped to SNOMED CT codes and even less for drug (25/556, 4.5%) and procedure concepts (8/214, 3.7%). Most used in EBMeDS were the ICD-10 and ICD-9 families, ICPC-2, ATC, and LOINC. For procedure, investigation result, and vaccine concepts, there appeared to be a lack of internationally accepted coding systems, since a lot of code sets included national coding systems or even electronic health record–specific proprietary coding systems.

There are several reasons for the popularity of the World Health Organization family of classifications. The comprehensiveness and widespread use of these classifications make them popular for clinical coding. The clinical codes in ICD are alphanumeric and arranged hierarchically. This allows for truncation of the codes in order to include a large number of child concepts with codes starting with the same sequence. For instance, by truncating the code K29*, it is possible to include the 10 different subcategories of gastritis and duodenitis without having to include each of these 10 codes. SNOMED CT, the other clinical coding system capable of representing a large majority of the clinical concepts, does not contain this feature because the unique identifiers of each code do not mirror the relationship to one another. This does not allow for truncation of the codes and explains why such a large number of codes are required to define each concept. As described earlier, ICD-10(-CM) only contains single parent-child relationships, but SNOMED CT includes multiple relationships. In investigating further, we found that if a clinical decision support system could recognize all possible SNOMED CT hierarchical relationships through a programmed expression, then the number of codes required to identify a concept was similar for SNOMED CT and ICD-10(-CM) (Multimedia Appendix 2). If, however, the clinical decision support system was only capable of recognizing the concept’s unique SNOMED CT identifier without its relationships, then a much larger number of codes was necessary. To date, very few SNOMED CT codes are included in EBMeDS mappings, and most mappings are for demonstration purposes only because the license to use SNOMED CT in EBMeDS has only recently been obtained. Therefore, the limited use of SNOMED CT may well be a consequence of fragmented uptake of this terminology in electronic health record systems that have integrated EBMeDS. ICPC-2, often used for documenting diseases and reasons for encounter in primary care, is of limited use in defining concepts required in clinical decision support systems; often the concepts defined in ICPC-2 are too broad and insufficiently detailed.

SNOMED CT has high sensitivity and specificity in representing clinical concepts [12,13]. However, creating code sets using SNOMED CT poses some important challenges. Through its poly-hierarchical structure, SNOMED CT creates an intricate web of clinical terms with multiple types of relationships defined through attributes. As opposed to a mono-hierarchical classification which has a branched structure, SNOMED CT has a profoundly complex web-like structure. This complexity may be a barrier to implementing this clinical coding system. In addition, SNOMED CT contains a very large number of terms, which makes it very difficult to create clinical code sets [8].

LOINC is very good at defining laboratory and physiological tests, but similar to that of SNOMED CT, the complex and granular classification structure is problematic. Despite the capacity of SNOMED CT to describe procedure codes, many countries use their own proprietary clinical coding systems. This may be as a result of locally used procedure lists generated for billing purposes which may not be internationally applicable. National or electronic health record system proprietary clinical coding systems may be very useful for some aggregate use of the electronic health record, but a myriad of coding systems for small-scale use requires multiple mappings and increases the odds of inappropriate mapping.

### Limitations

For this study, we chose to limit the number of recommendations that we analyzed for each of the coding systems or terminologies. Manual searching of codes and terms that applied to individual clinical concepts proved time consuming and is, therefore, not feasible for a larger set of recommendations. We also limited the clinical coding systems to those systems currently in use in Europe. Therefore, several clinical coding systems that were included in the practical evaluation were not included in the theoretical evaluation, such as Current Procedural Terminology. The code sets were constructed by one person (ND) and were not validated by a second reviewer. The small scale of this first assessment and the lack of external validation of the code sets demands caution when drawing conclusions, but some trends were clear. We correlated these trends with a large EBMeDS database of existing mappings used in an internationally available clinical decision support system. The use of clinical coding systems in EBMeDS may not necessarily imply that they are well suited for defining concepts but may merely mirror the de facto use of these coding systems in the electronic health records where EBMeDS is integrated.

Our study did not assess all possible domains that may need translation into clinical coding. We did not study any terminologies or classifications that attempt to structure concepts such as pain, distress, anxiety, or other more complicated concepts. These types of clinical information are currently often lacking in clinical decision support systems and remain underexposed in studies.

In addition, the findings from this study are limited to one particular aspect of clinical decision support systems, namely the efficiency of particular clinical coding systems in correctly defining clinical concepts required to translate recommendations in decision support rules. Our study does not evaluate the efficiency of these systems in assisting clinicians in high-quality documentation at the point of care. More important than the capacity of a clinical coding system to correctly define a clinical concept may be the capacity of clinicians to correctly use these systems to document clinical data into the electronic health record. In a study [14] on clinical decision support systems using gastrointestinal risk scores, when confronted with identical patients consulting for identical problems, differences in how clinical information was recorded led to almost 80% of inaccurate recommendations by the clinical decision support systems. Similarly, a recent study [15] on clinical decision support system alerts on potential adverse drug events showed that almost 9 out of 10 alerts were overridden and that more than 8 out of 10 of these overrides were appropriate [15]. Quite often, alerts were triggered on drugs that had been stopped but were inadequately documented in the electronic health record. These findings suggest that the true bottleneck in data quality is probably not due to limitations in data coding or terminologies but to the quality of the documentation by clinicians or other sources of bias in electronic health records [16].

### Implications

One would expect that the more detailed a coding system becomes, the more suited it becomes for defining concepts necessary for clinical decision support systems. However, it is unclear whether SNOMED CT, currently the most comprehensive clinical coding system available, is also the best choice for developing clinical decision support system artifacts. Through its rich poly-hierarchical relationships, SNOMED CT is growing into a true ontology potentially allowing for consistent documentation of practically all aspects of health care [17]. A European comprehensive evaluation of SNOMED CT implementations recognized the pivotal role of SNOMED CT as a core reference terminology but placed it as a part of a greater ecosystem of terminologies [18]. Important advantages of SNOMED CT are its single ownership, unique source, and clear ontology-based architecture, including the capacity to postcoordinate (combine concepts to create new, more detailed concepts). This potential is offset by important disadvantages such as its complexity and granularity, which require a comprehensive understanding of its structure before it can be used for knowledge representation in a clinical decision support system. Moreover, SNOMED CT still needs to prove its usability and user-friendliness as a clinical coding dictionary at the point of care, since its comprehensiveness may very well be a burden rather than an advantage as illustrated in the satirical paper by Richard Williams [19]. Hence, clinical coding systems suited for clinical documentation may not necessarily be the most adequate for information retrieval or other secondary use such as clinical decision support systems. A possible solution to this problem could be the development of local vocabularies that contain clinical terms which are mapped to multiple clinical coding systems, including reference terminologies such as SNOMED CT [10,20]. This would allow clinicians, and other potential users, to code clinical information using routinely used terms, simultaneously documenting the data in multiple structures. Depending on the type of aggregate use, clinical decision support system, quality-of-care indicator measurement, pay-for-performance schemes, or health policies, different clinical codes can be queried in the electronic health record.

### Conclusions

Translating recommendations from clinical guidelines into artifacts for clinical decision support systems is an important step in implementing evidence-based health care. Not all clinical coding systems used in electronic health records for routine collection of clinical data are equally efficient in defining the concepts in clinical decision support system artifacts. Research is needed to study whether the use of more comprehensive clinical coding systems such as SNOMED CT influences the appropriateness of clinical decision support system alerts.

## Appendix

Multimedia Appendix 1 Preventive care recommendations.

Multimedia Appendix 2 Code sets.

## Abbreviations

ATC: Anatomical Therapeutic Chemical Classification
EBMeDS: Evidence-Based Medicine electronic Decision Support
ICD-10: International Classification of Diseases, Tenth Revision
ICPC: International Classification of Primary Care
LOINC: Logical Observation Identifiers Names and Codes
SNOMED CT: Systematized Nomenclature of Medicine—Clinical Terms
