# Supplementary material for: Coding Systems for Clinical Decision Support: Theoretical and Real-World Comparative Analysis
Source: JMIR Form Res. 2020 Oct 21;4(10):e16094. doi: 10.2196/16094 (PMC7641774; doi:10.2196/16094)
Supplement: Multimedia Appendix 1 [file formative_v4i10e16094_app1.pdf]

Appendix 1

Preventive care recommendations used in the theoretical evaluation of coding systems for clinical decision support.

Recommendation 1

Pneumococcal vaccination for patients with an elevated risk of pneumococcal sepsis
The Domus Medica Health Guide advises that people with an elevated risk for pneumococcal sepsis be vaccinated with a combination of the 23 –valent saccharide and the 13-valent conjugated pneumococcal vaccines. Patients are considered to have an elevated risk if they:

- Have a functional or anatomical asplenia, sickle cell disease or a hemoglobinopathy
- Have a cerebrospinal fluid leak or a cochlear implant
- Have a weakened immunity (such as hematological malignancies, immunosuppressive medication)

The vaccination course consists of a primary vaccination and booster vaccinations.
The primary vaccination: PCV13 followed by PPV23 after 8 weeks
Booster vaccinations: PPV23 every 5 years
The Domus Medica Health Guide refers to the guidelines of the High Commission for Health Care (Hoge Gezondheidsraad), but clearly states that there is little evidence for the efficacy of the proposed vaccination course in preventing pneumococcal sepsis in patients considered having an elevated risk.

Recommendation 2

Three yearly opportunistic screening for diabetes type 2 is advised for persons aged 45 to 64 years with at least one of the following risk factors:

- History of giving birth to a baby weighing more than 4.5 kg,
- Relative in the first degree with diabetes type 2,
- BMI ≥ 25,
- Abdominal circumference of > 88 cm (women) or > 102 cm (men),
- Hypertension,
- Treatment with corticoids.

Screening on a yearly basis is recommended for all persons:

- Aged more than 65 years,
- History of gestational diabetes,
- History of stress hyperglycemia (documented during an intervention or hospitalization),
- With an elevated fasting blood glucose or elevated glucose tolerance test.

Recommendation 3

Two yearly screening for breast cancer is advised for women between 50 and 69 years of age without a family history of breast cancer. After the age of 70 years, the choice whether to continue the screening should be discussed with the patient.
