# Supplementary material for: Coding Systems for Clinical Decision Support: Theoretical and Real-World Comparative Analysis
Source: JMIR Form Res. 2020 Oct 21;4(10):e16094. doi: 10.2196/16094 (PMC7641774; doi:10.2196/16094)
Supplement: Multimedia Appendix 2 [file formative_v4i10e16094_app2.pdf]

Recommendation 1

Pneumococcal vaccination for patients with an elevated risk of pneumococcal sepsis

The Domus Medica Health Guide advises that people with an elevated risk for pneumococcal sepsis be vaccinated with a combination of the 23 –valent saccharide and the 13-valent conjugated pneumococcal vaccines. Patients are considered to have an elevated risk if they:

- Have a functional or anatomical asplenia, sickle cell disease or a hemoglobinopathy
- Have a cerebrospinal fluid leak or a cochlear implant
- Have a weakened immunity (such as hematological malignancies, immunosuppressive medication)

The vaccination course consists of a primary vaccination and booster vaccinations.

The primary vaccination: PCV13 followed by PPV23 after 8 weeks

Booster vaccinations: PPV23 every 5 years

Containers

Diagnoses

There are several diagnoses which need to be coded for this guideline to be written into a script. The group of patients identified as having an elevated risk of invasive pneumococcal infections are:

- Patients with a functional or anatomic asplenia
- Sickle cell disease
- hemoglobinopathy

Asplenia

ICPC-2

ICPC2 doesn’t have a code for asplenia. It has a code B99 (Blood/lymph/spleen disorder other). In decision support rules it is preferred to avoid unspecific codes since this may generate reminders in situations where this is not intended.

ICD-10

ICD-10 contains two possible codes for asplenia.

- Asplenia (congenital): Q89.01
- Status (post) splenectomy: Z90.81

SNOMED CT

SNOMED CT has 2 codes pertaining to asplenia.

- Congenital asplenia: 93030006

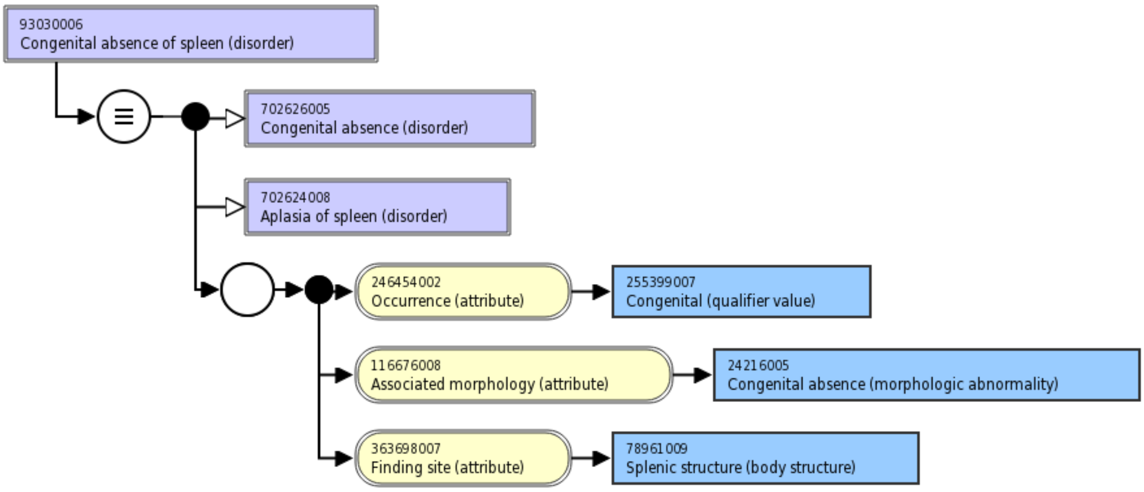

- Functional asplenia (disorder): 38096003

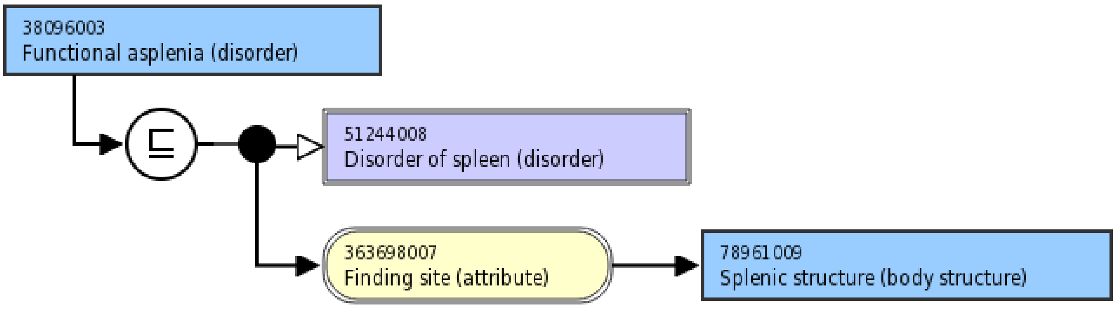

Sickle cell disease

ICPC-2

ICPC2 doesn't have a code for sickle cell disease. It has a code B79 (Congenital anomaly blood/lymph other) or the code B78 (hereditary hemolytic anemia). These codes are however too unspecific for use in clinical decision support.

ICD-10

ICD-10 contains code(s) for sickle cell disease.

- Sickle-cell disorders: D57

SNOMED CT

SNOMED CT has a code for sickle cell disease.

- Sickling disorder due to hemoglobin S (disorder)

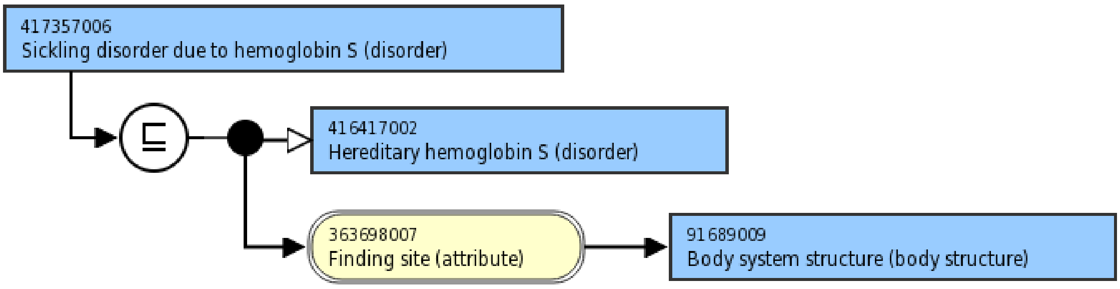

Hemoglobinopathy

ICPC-2

ICPC2 doesn't have a code for hemoglobinopathy. It has a code B78 (Hereditary hemolytic anaemia), B79 (Congenital anomaly blood/lymph other) and B82 (Anemia other/unspecified). These codes are however too unspecific for use in clinical decision support.

ICD-10

ICD-10 contains code(s) for thalassemia's.

- Thalassemia: D56

SNOMED CT

SNOMED CT has a code that includes all forms of hemoglobinopathy.

- Hemoglobinopathy (disorder): 80141007

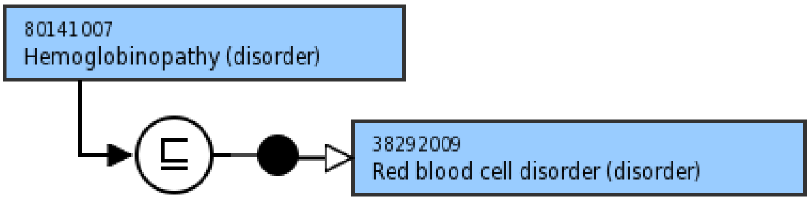

Note that the SNOMED CT code hemoglobinopathy has 10 "children", including "hereditary hemoglobinopathy (disorder)" which in turn has 5 "children", including "homozygous hemoglobinopathy (disorder)" which in turn is the parent of "hereditary hemoglobinopathy disorder homozygous for hemoglobin S (disorder)". This last code is also the parent of the code "Sickling disorder due to hemoglobin S (disorder)". These links imply that the code "Hemoglobinopathy (disorder)" includes patients with sickle cell disease.

CSF leak

ICPC-2

There is no ICPC-2 code specifically for classifying a CSF leak. There exists a code "injury nervous system other": N81. This code, however is too unspecific for use in this decision support rule.

ICD-10

There are two codes that classify a cerebrospinal fluid leak.

- Cerebrospinal fluid leak (excl. from spinal puncture): G96.0
- Cerebrospinal fluid leak from spinal puncture: G97.0

It is unclear from the description of the evidence base whether both forms of cerebrospinal fluid leak are to be included as patients with an elevated risk, but given that a CSF leak from spinal puncture is mostly a self-limiting problem, we assume that the first option is the code we need.

SNOMED CT

SNOMED CT contains a description for cerebrospinal fluid leak.

- Cerebrospinal fluid leak (disorder): 230744007

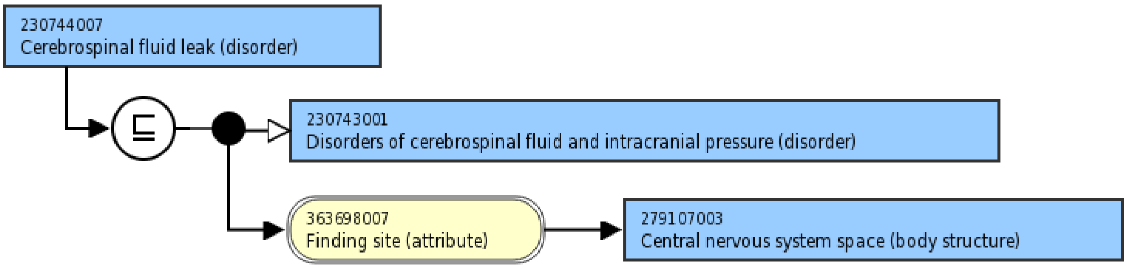

Cochlear implant

ICPC-2

There is no code for cochlear implant in ICPC-2.

ICD-10

ICD-10 knows one code for cochlear implants.

- Presence of otological and audiological implants (incl. bone-conduction hearing device, cochlear implant, Eustachian tube stent, myringotomy tube(s), stapes replacements): Z96.2

This code will also include all patients with myringotomy tubes as being at risk. There is a risk of over diagnosing people with cochlear implants using the code Z96.2.

SNOMED CT

SNOMED CT contains several descriptions pertaining to cochlear implants, where the most relevant is the identifier within the "physical object" container.

- Cochlear prosthesis, device (physical object): 43252007

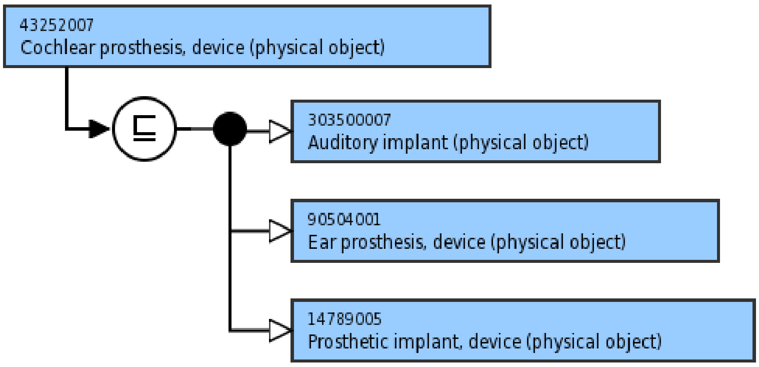

Weakened immunity

ICPC-2

There is no ICPC-2 code for patients with a weakened immunity. There is no code for patients using immunosuppressants. There are three codes classifying patients with hematological malignancies.

- Hodgkin's disease/lymphoma: B72
- Leukemia: B73
- Malignant neoplasm blood other: B74

ICD-10

ICD-10 classifies disorders involving the immune system under a series of codes.

- Certain disorders involving the immune system: D8* (where * can be any digit or series of digits)
    - Immunodeficiency with predominantly antibody defects: D81
    - Combined immunodeficiencies: D81
    - Immunodeficiency associated with other major defects: D82
    - Common variable immunodeficiency: D83
    - Other immunodeficiencies: D84
    - Sarcoidosis: D86
    - Other disorders involving the immune mechanism, not elsewhere classified: D89

ICD-10 also classifies neoplasms of hematopoietic or lymphoid tissue under a series of codes.

- Malignant neoplasms, stated or presumed to be primary, of lymphoid, hematopoietic and related tissue: C81-C96
    - Hodgkin lymphoma: C81
    - Follicular lymphoma: C82
    - Non-follicular lymphoma: C83
    - Mature T/NK cell lymphomas: C84
    - Other and unspecified types of non-Hodgkin lymphoma: C85
    - Other specified types of T/NK cell lymphomas: C86
    - Malignant immunoproliferative diseases: C88
    - Multiple myeloma and malignant plasma cell neoplasms: C90
    - Lymphoid leukemia: C91
    - Myeloid leukemia: C92
    - Monocytic leukemia: C93
    - Other leukemias of specified cell type: C94
    - Leukemia of unspecified cell type: C95
    - Other and unspecified malignant neoplasms of lymphoid, hematopoietic and related tissue: C96

SNOMED CT

SNOMED CT contains a concept including all patients with a disorder of the immune function.

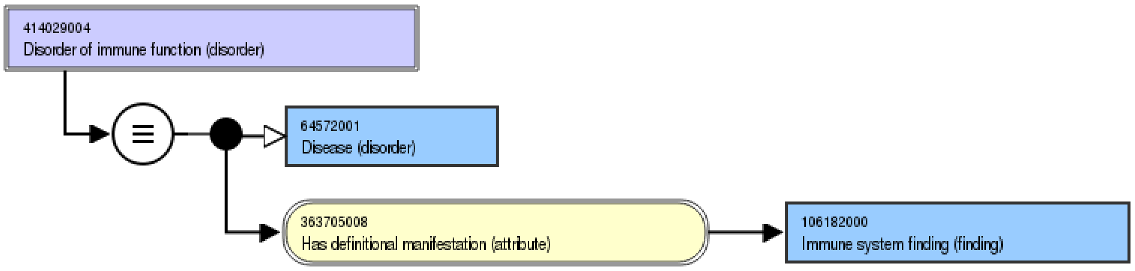

The SNOMED CT concept “Disorder of the immune function” includes the concept including patients with an immunodeficiency disorder.

- Immunodeficiency disorder (disorder): 234532001

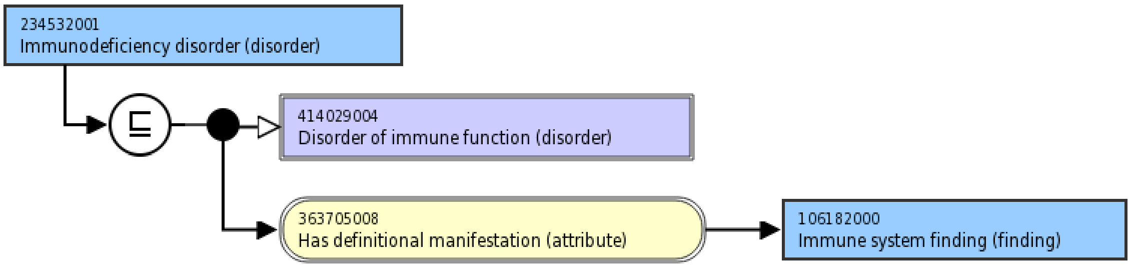

It also includes a concept including patients with a hematological neoplasm.

- Hematologic neoplasm (disorder): 129154003

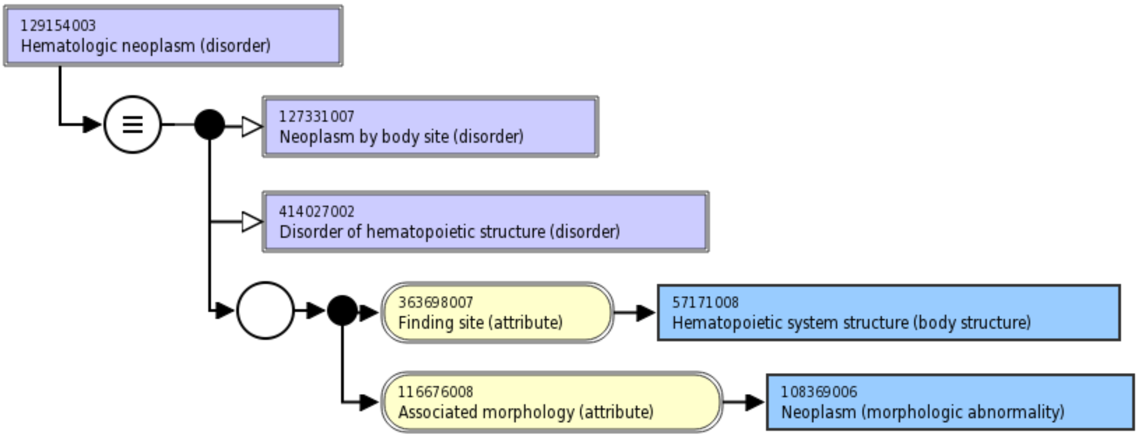

SNOMED CT also contains several descriptions for patients undergoing immunosuppressant therapy. For instance:

- Patient immunosuppressed (finding): 370391006

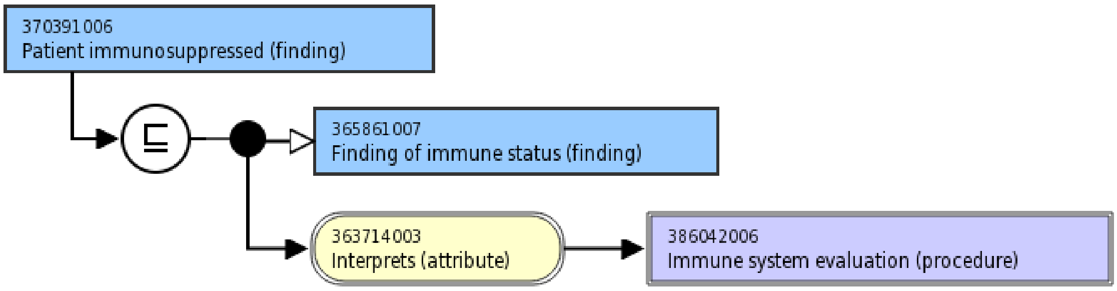

Drugs

Immunosuppressant

ATC

- Immunosuppressants: L04A

SNOMED CT

- Immunosuppressant (product): 69431002

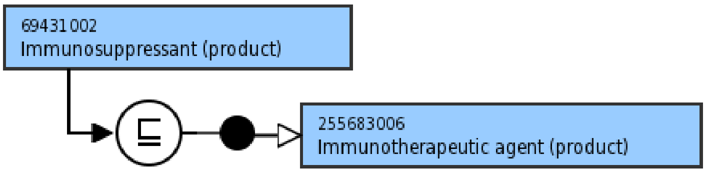

Vaccinations

For the correct function of the clinical decision support rule, the decision support application must be able to "read" the date of administration of the vaccine in the EHR. This prerequisite is independent of the used coding system but is a part of the clinical document structure (CDA) used within the EHR. Discussion of CDA is beyond the scope of this use case.

Pneumococcal polysaccharide vaccine (23-valent)

ATC

- pneumococcus, purified polysaccharides antigen: J07AL01

SNOMED CT

- pneumococcal polysaccharide vaccine (product): 135642004

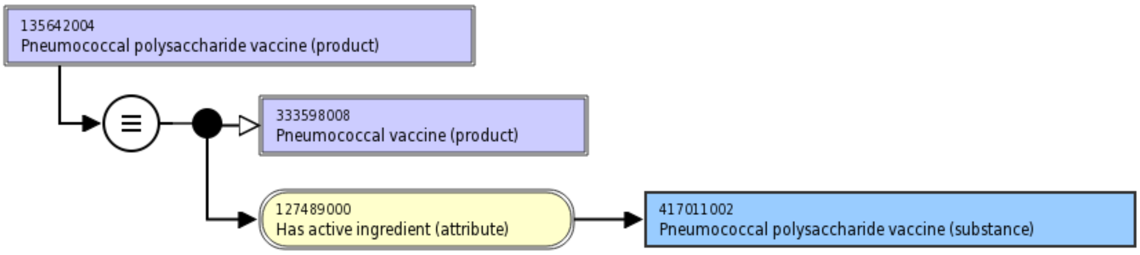

Pneumococcal 13-valent conjugate vaccine

ATC

- pneumococcus, purified polysaccharides antigen conjugated: J07AL02

SNOMED CT

- Pneumococcal 13-valent conjugate vaccine (product): 448964007

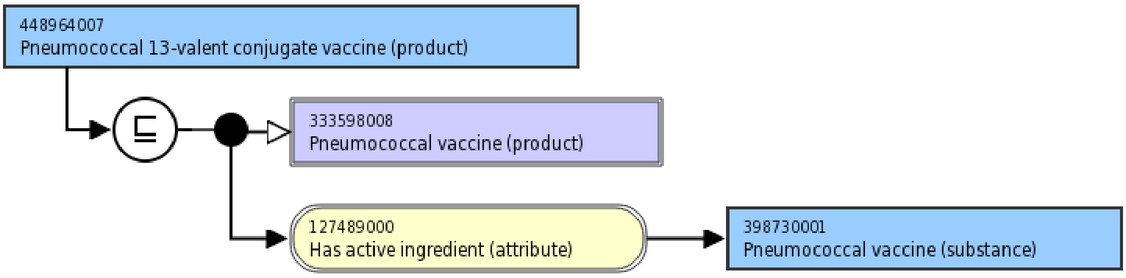

Recommendation 2

Three yearly opportunistic screening for diabetes type 2 is advised for persons aged 45 to 64 years with at least one of the following risk factors:

- History of giving birth to a baby weighing more than 4.5 kg,
- Relative in the first degree with diabetes type 2,
- BMI ≥ 25,
- Abdominal circumference of > 88 cm (women) or > 102 cm (men),
- Hypertension,
- Treatment with corticoids.

Screening on a yearly basis is recommended for all persons:

- Aged more than 65 years,
- History of gestational diabetes,
- History of stress hyperglycemia (documented during an intervention or hospitalization),
- With an elevated fasting blood glucose or elevated glucose tolerance test.

Containers

Diagnoses

The development of this script requires certain coded diagnoses. More specifically, patients with hypertension will need to be identified.

Hypertension

ICPC-2

Hypertension can be coded in two ways using ICPC-2:

- K86 (Hypertension uncomplicated) or
- K87 (Hypertension complicated).

ICD-10

ICD-10 contains five different classes for hypertension:

- I10: Essential (primary) hypertension
- I11: Hypertensive heart disease
- I12: Hypertensive renal disease
- I13: Hypertensive heart and renal disease
- I15: Secondary hypertension

By truncating, using ICD-10, hypertension can be coded as I1*.

SNOMED CT

Within SNOMED CT hypertension is coded as 38341003.

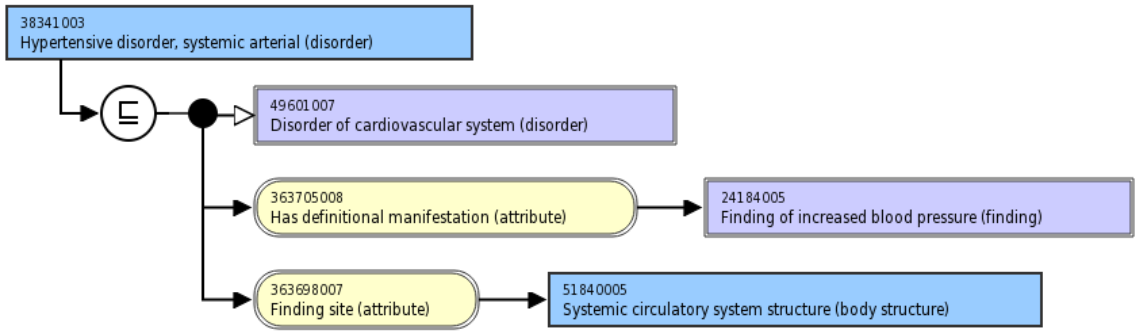

The code 38341003 has 24 children including:

- Benign hypertension (disorder)
- Diastolic hypertension (disorder)
- Eclampsia added tot pre-existing hypertension (disorder)
- Essential hypertension (disorder)
- Exertional hypertension (disorder)
- Hypertension complicating pregnancy, childbirth and the puerperium (disorder)
- Hypertension in chronic kidney disease due to type 2 diabetes mellitus (disorder)
- Secondary hypertension (disorder)
- …

Using the post coordinated codes of the "child" codes would adequately be able to identify all possible concepts including the code for hypertension.

Family history

The container for recording the family history must be properly identified as diagnoses for family members and not for the patient himself. This implies that all the items in this container are recognized as "family history" through the way it is structured.

Diabetes type 2

ICPC-2

Type 2 diabetes can be coded using the following:

- T90: Diabetes non-insulin dependent

Although the description of the code is "non-insulin dependent" diabetes, in daily use, the code is used to identify patients with type 2 diabetes.

ICD-10

Type 2 diabetes can be coded using the following:

- E11: Type 2 diabetes mellitus

This code includes the following child codes:

- E11.0: with coma
- E11.1: with ketoacidosis
- E11.2: with renal complications
- E11.3: with ophthalmic complications
- E11.4: with neurological complications

- E11.5: with peripheral circulatory complications
- E11.6: with other specified complications
- E11.7: with multiple complications
- E11.8: with unspecified complications
- E11.9: without complications

SNOMED CT

SNOMED CT allows for coding family histories. Even though it is still important that these elements are correctly structured within the EHR, these codes are different from the “disorder” codes making it easier to recognize them as a family history element.

A family history for diabetes type 2 can be coded using the following concept:

- 430679000: Family history of diabetes mellitus type 2 (situation)

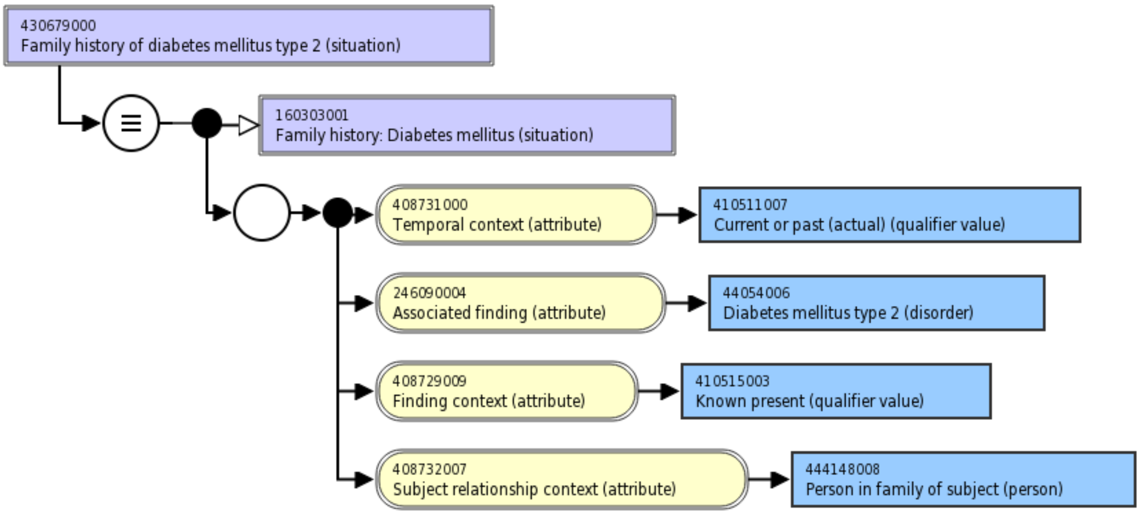

Medical history

The difficulty is identifying a medical history is in the choice where to document this item in the EHR. Traditionally, all relevant diagnoses are documented in a diagnosis/problem list. Defining what is “relevant” and what is not is sometimes rather arbitrary. Is giving birth to a child of 5 kg a relevant diagnosis?

Besides the question of defining active problems, there is also the problem of how these items are structured in the EHR. Some EHRs define “medical history” as all those problems which have been marked as “past” or “inactive”. Other EHRs have a separate container for “medical history” not linked to the problem list. Using existing archetypes for medical history and the corresponding EN13606, *open*EHR or HL7 definitions is necessary to enable communication with the decision support service.

Gestational diabetes

ICPC-2

Gestational diabetes can be coded using the following:

- W85: Gestational diabetes

ICD-10

Gestational diabetes can be coded using the following:

- O24.4: Diabetes mellitus arising in pregnancy (Gestational diabetes mellitus NOS)

SNOMED CT

SNOMED CT allows for the coding of medical history, different from active “disorders”. A history of gestational diabetes can be coded using the following:

- 472971004: History of gestational diabetes mellitus (situation)

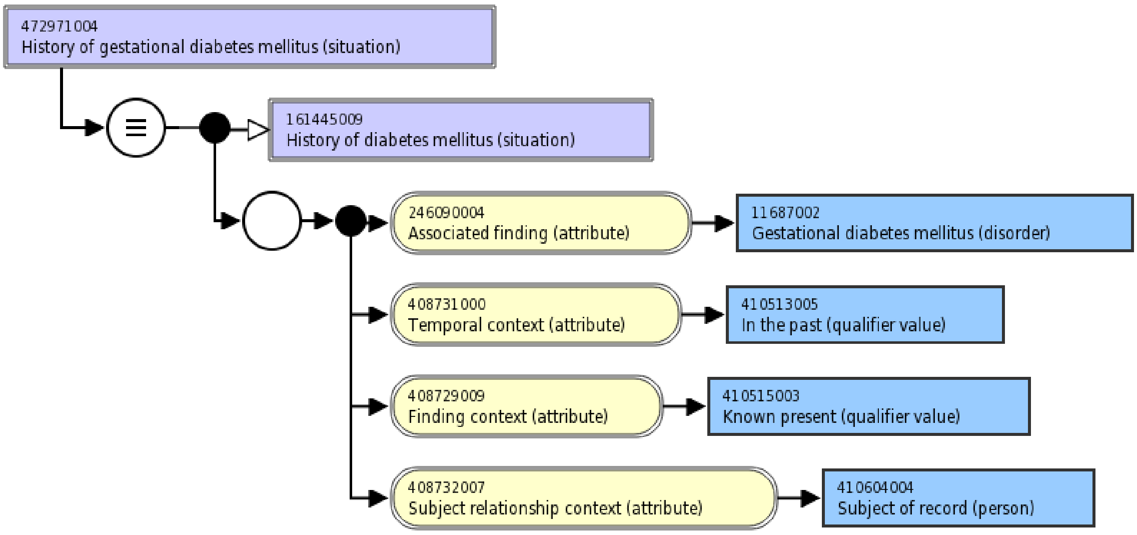

- 11687002: Gestational diabetes mellitus (disorder)

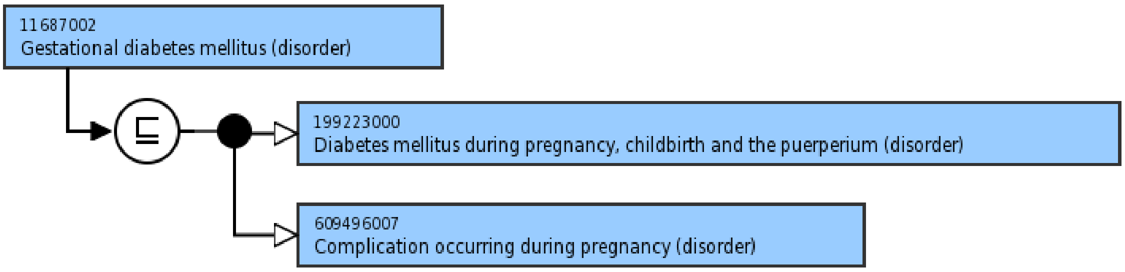

Using post coordinated codes, it is possible to identify all patients with (a history of) gestational diabetes using only the code 11687002 because the pre-coordinated code 472971004 can be translated into a set of post coordinated codes including 11687002.

Stress hyperglycemia

ICPC-2

ICPC-2 has no code for stress hyperglycemia

ICD-10

ICD-10 allows for the coding of an elevated blood glucose level, however without identifying this as a “stress hyperglycemia”.

- R73: Elevated blood glucose level
    - R73.0: Abnormal glucose tolerance test
    - R73.9: Hyperglycemia, unspecified

SNOMED CT

Stress hyperglycemia can be coded as:

- 237624007: Metabolic stress hyperglycemia (disorder)

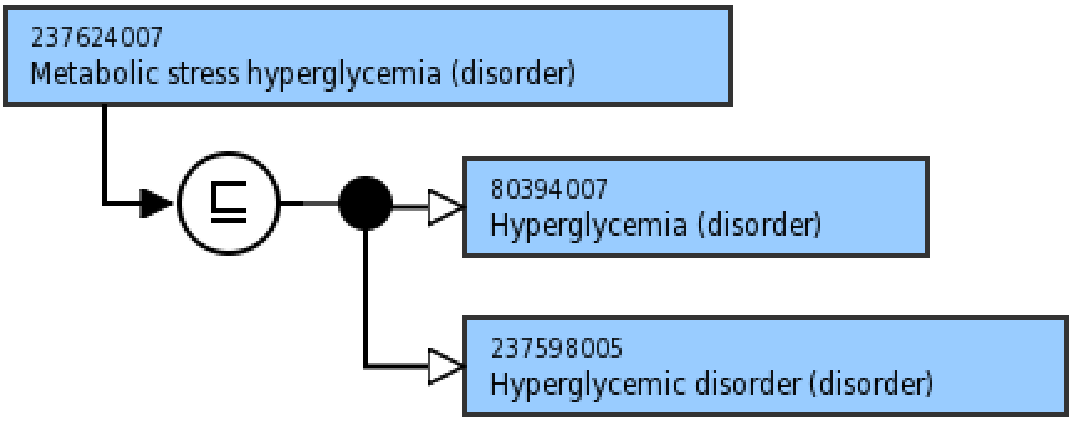

Baby > 4.5 kg

ICPC-2

ICPC-2 doesn't allow for coding elevated birth weight.

ICD-10

High birth weight can be coded as:

- P08.0: Exceptionally large baby (usually implies a birth weight of 4500 g or more)

SNOMED CT

High birth weight can be coded as:

- 276613009: High birth weight (disorder)

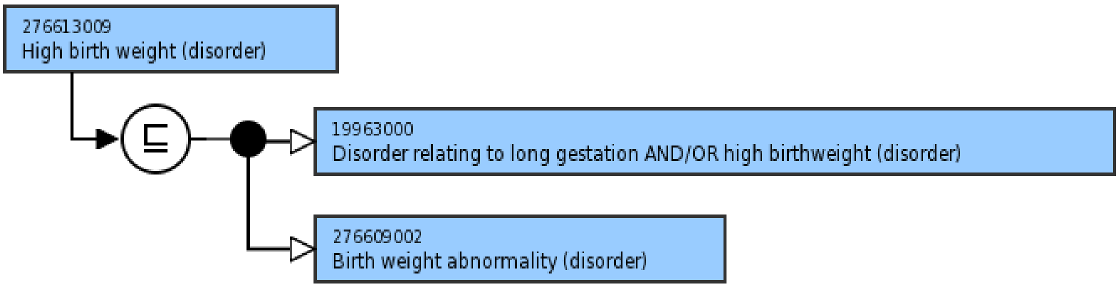

The code 276613009 has 2 child codes:

- 38206000: Exceptionally large at birth (disorder)
- 7293009: Heavy-for-dates at birth regardless of gestation period (disorder)

SNOMED CT has no concept for "history of high birth weight".

Procedures

Abdominal circumference

ICPC-2

There are very few procedure codes in ICPC-2. ICPC-2 has the following codes for physical examination:

- *-30: Medical Exam/Eval-Complete
- *-31: Medical Examination/Health Evaluation-Partial/Pre-op check

These codes are not specific enough to code for abdominal circumference.

ICD-10

Neither ICD-10 nor ICD-10 CM contains a code for abdominal circumference.

SNOMED CT

Abdominal circumference can be coded as:

- 396552003: Abdominal circumference (observable entity)

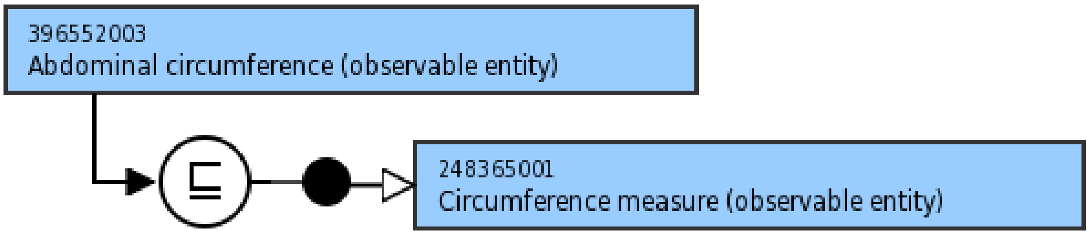

LOINC

LOINC has several codes for abdominal circumference:

- 56115-9: Waist circumference by NCFS (in cm),
- 56114-2: Waist circumference by NHANES (in cm),
- 56117-5: Waist circumference by WHI (in cm),
- 8280-0: Waist circumference at umbilicus by tape measure (in cm),
- 8281-8: Waist circumference at umbilicus by US (in cm).

Body Mass Index

ICPC-2

Besides the codes mentioned above, ICPC-2 has no specific code for BMI.

ICD-10

ICD-10 CM has several codes for BMI:

- Z68: Body mass index (BMI), including:
  - Z68.1: Body mass index (BMI) 19 or less, adult
  - Z68.2: Body mass index (BMI) 20-29, adult
    - Z68.20: Body mass index (BMI) 20.0-20.9, adult
    - …
  - Z68.3: Body mass index (BMI) 30-39, adult
    - Z68.30: Body mass index (BMI) 30.0-30.9, adult

        - …
    - Z68.4: Body mass index (BMI) 40-49, adult
        - Z68.40: Body mass index (BMI) 40.0-40.9, adult
        - …
    - Z68.5: Body mass index (BMI) pediatric
        - Z68.50: Body mass index (BMI) pediatric less than 5th percentile for age
        - …

SNOMED CT

BMI can be coded as:

- 60621009: Body mass index (observable entity)

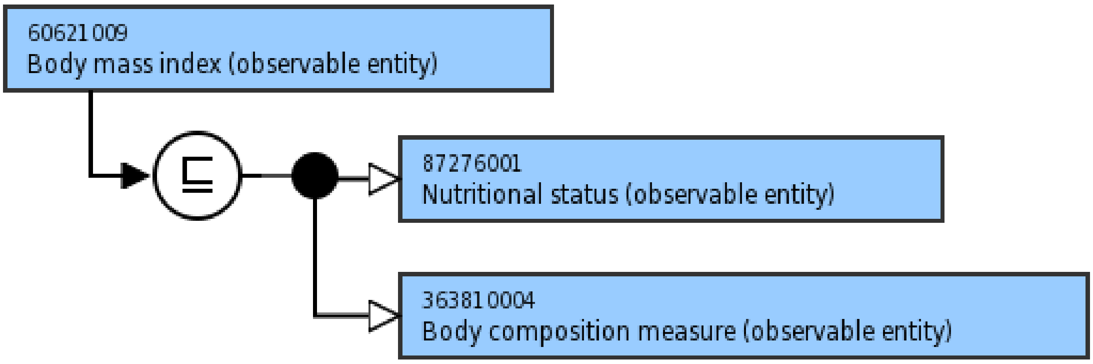

LOINC

BMI can be coded as:

- 74728-7: Vital signs, weight, height, head circumference, oximetry, BMI and BSA panel – HL7
    - 39156-5: Body mass index (BMI) (in kg/m2)

Drugs

Corticosteroids

Corticosteroids in this use case can be specified as glucocorticoids for systemic use.

ATC

Glucocorticoids for systemic use can be coded using ATC as follows:

- H02AB: Glucocorticoids

This class of drugs includes:

- H02AB01: betamethasone
- H02AB02: dexamethasone
- H02AB03: fluocortolone
- H02AB04: methylprednisolone
- H02AB05: paramethasone
- H02AB06: prednisolone
- H02AB07: prednisone
- …

By truncating the code to H02AB*, all the underlying codes can be included in the definition of the concept.

SNOMED CT

Glucocorticoids can be coded using SNOMED CT as follows:

- 116569008: Synthetic glucocorticoid (substance)

This code includes 9 children, including all the drugs as described under the ATC code H02AB*.

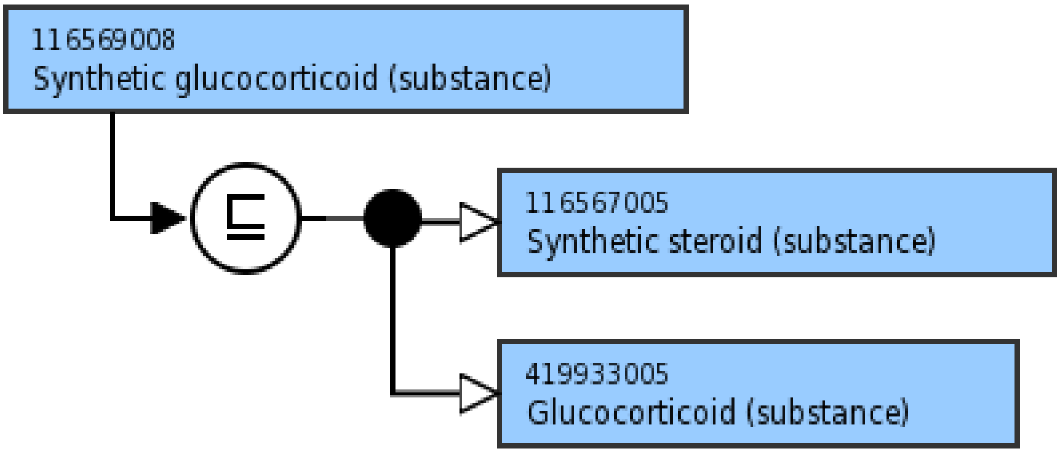

From the description of the code, it is however unclear whether topical substances are included or only systemic substances. The inclusion and exclusion criteria for glucocorticoids are more clearly stated in the ATC classification.

Laboratory tests

Fasting blood glucose

LOINC

Fasting glucose in venous blood can be coded as:

- 1557-8: Fasting glucose [Mass/volume] in venous blood (in mg/dL)
- 1558-6: Fasting glucose [Mass/volume] in serum or plasma (in mg/dL)
- 14771-0: Fasting glucose [Mass/volume] in serum or plasma (in mmol/L)
- 17865-7: Glucose [Mass/volume] in serum or plasma – 8 hours fasting (in mg/dL)
- 10450-5: Glucose [Mass/volume] in serum or plasma – 10 hours fasting (in mg/dL)
- 1554-5: Glucose [Mass/volume] in serum or plasma – 12 hours fasting (in mg/dL)
- 53049-3: Glucose [Mass/volume] – pre-meal (in mg/dL)

SNOMED CT

Fasting glucose in venous blood can be coded as:

- 271062006: Fasting blood glucose measurement (procedure)

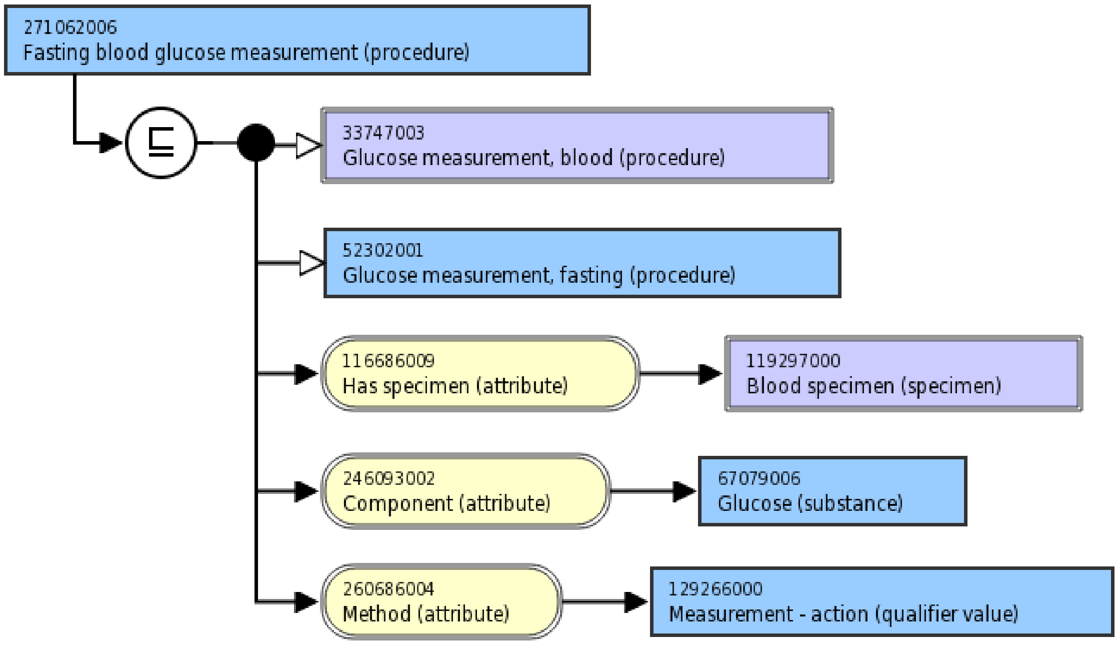

OGTT

The WHO has defined the oral glucose tolerance test (OGTT) with the following procedure:

- A baseline fasting blood glucose sample at time zero
- The patient is then given a measured glucose sample to drink (the dose can vary but is often between 75 and 100g of glucose)
- (Optional) a blood glucose sample at time 1 hour
- A blood glucose sample at time 2 hours
- (Optional) additional blood glucose samples up to 6 hours

LOINC

The OGTT can be coded as:

- 24353-5: Glucose tolerance 2 hours gestational panel - Urine and Serum or Plasma, including:
  - 2345-7: Glucose [Mass/volume] in Serum or Plasma (in mg/dL)
  - 20438-8: Glucose [Mass/volume] in Serum or Plasma --1 hour post dose glucose (in mg/dL)
  - 20436-2: Glucose [Mass/volume] in Serum or Plasma --2 hours post dose glucose (in mg/dL)
- 72171-2: Glucose tolerance 2 hours panel - Serum or Plasma
  - 14771-0: Fasting glucose [Mass/volume] in serum or plasma (in mmol/L)
  - 25665-1: Glucose [Moles/volume] in Serum or Plasma --1 hour post XXX challenge (in mmol/L)
  - 25679-2: Glucose [Moles/volume] in Serum or Plasma –1.5 hours post XXX challenge (in mmol/L)
  - 25668-5: Glucose [Moles/volume] in Serum or Plasma --2 hours post XXX challenge (in mmol/L)

There exist at least another 20 different codes for glucose challenge or tolerance tests specifying either the dose of the glucose sample or the time of the blood sample. There are also codes that

define a post-prandial (after meal) glucose test. To avoid false negative or false positive reminders, we advise the use of true OGTT or challenge codes that specify a test with a measured dose of glucose.

SNOMED CT

The OGTT can be coded as:

- 113076002: Glucose tolerance test (procedure), including 1 child
    - 699731004: Glucose tolerance test, antenatal (procedure)

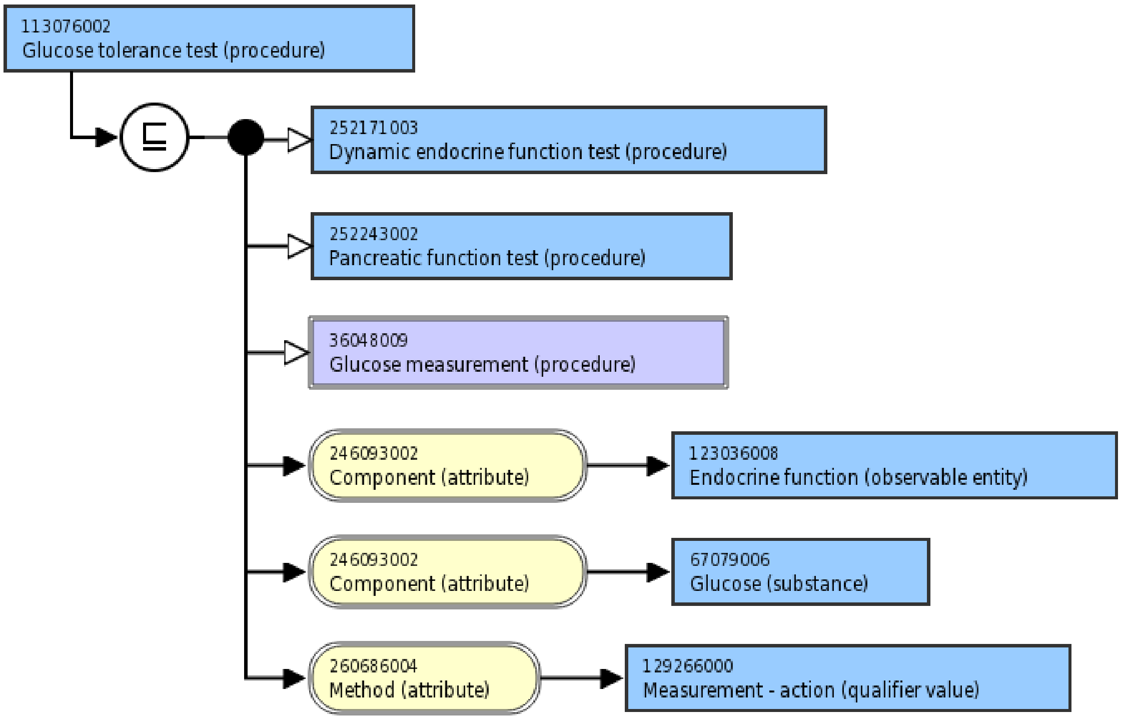

The SNOMED CT concept only specifies that the OGTT is a “measurement of blood glucose in the fasting state and at specific intervals before and after oral or intravenous glucose load”. It doesn’t specify the glucose dose nor the time intervals. This could lead to many different values defined using the same concept but collected under different circumstances.

Recommendation 3

Two yearly screening for breast cancer is advised for women between 50 and 69 years of age without a family history of breast cancer. After the age of 70 years, the choice whether to continue the screening should be discussed with the patient.

Containers

Family history

Breast cancer

ICPC-2

A family history of breast cancer can be coded using ICPC-2 assuming that the code is documented under the correct structured element of archetype for family history within the EHR. Breast cancer can be coded using:

- X76: Malignant neoplasm breast female

ICD-10

A family history of breast cancer can be coded using ICD-10 assuming that the code is documented under the correct structured element of archetype for family history within the EHR. Breast cancer can be coded using:

- C50: Malignant neoplasm of breast, including:
  - C50.0: Nipple and aureola
  - C50.1: Central portion of breast
  - C50.2: Upper-inner quadrant of breast
  - C50.3: Lower-inner quadrant of breast
  - C50.4: Upper-outer quadrant of breast
  - C50.5: Lower-outer quadrant of breast
  - C50.6: Axillary tail of breast
  - C50.8: Overlapping lesion of breast
  - C50.9: Breast, unspecified

SNOMED CT

A family history of breast cancer can be coded by the following codes:

- 429740004: Family history of malignant neoplasm of breast (situation), including
  - 430292006: Family history of malignant neoplasm of breast in first degree relative (situation)
  - 428748002: Family history of malignant neoplasm of male breast (situation)

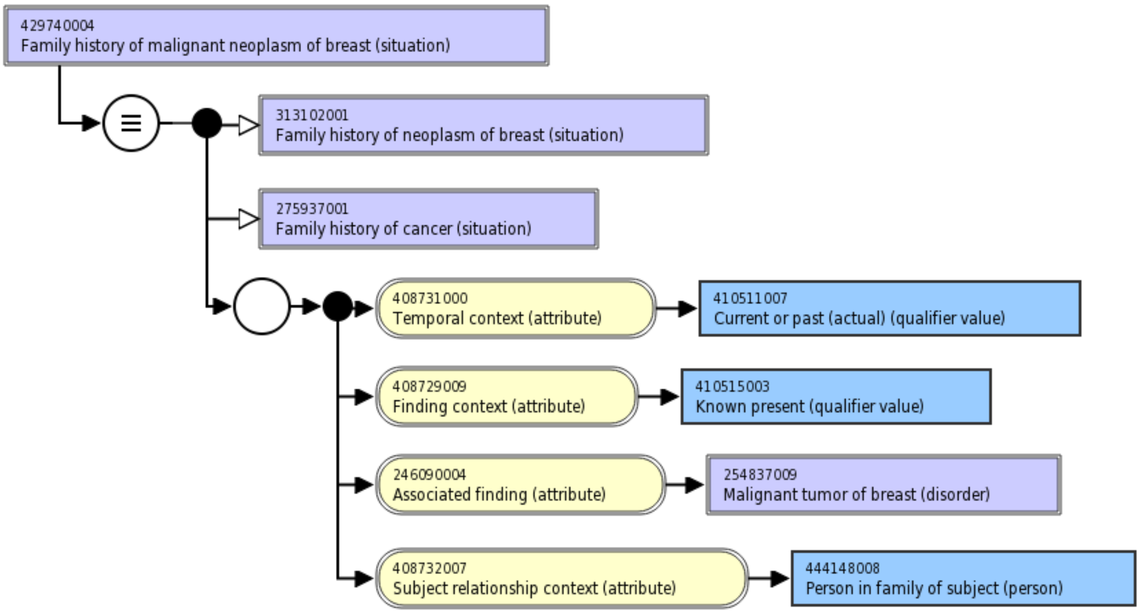

Additionally, a family history for BRCA1 or BRCA2 can also be coded using the following:

- 431330007: Family history of breast cancer 1 (BRCA1) gene mutation (situation)
- 431330006: Family history of breast cancer 2 (BRCA2) gene mutation (situation)

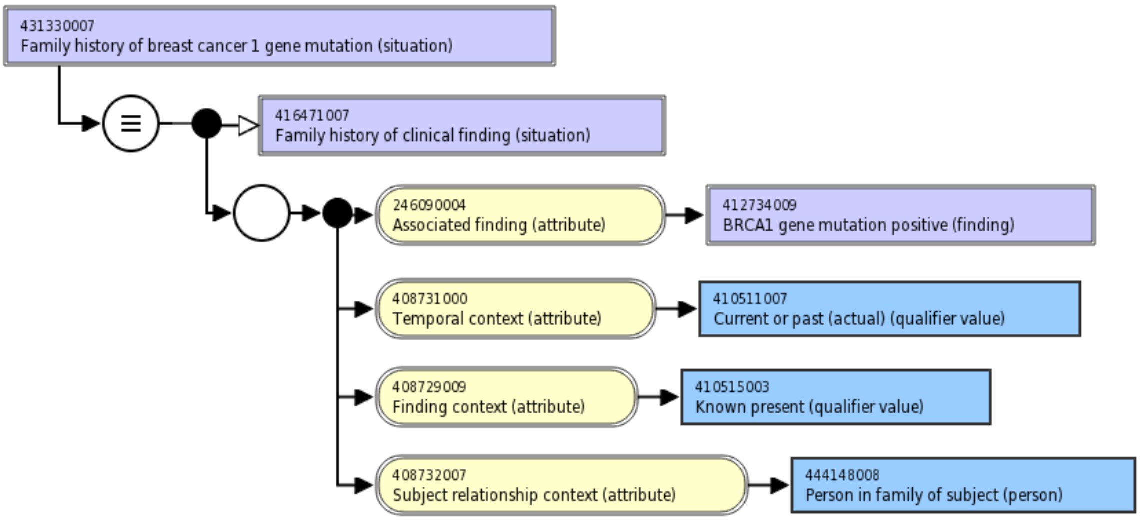

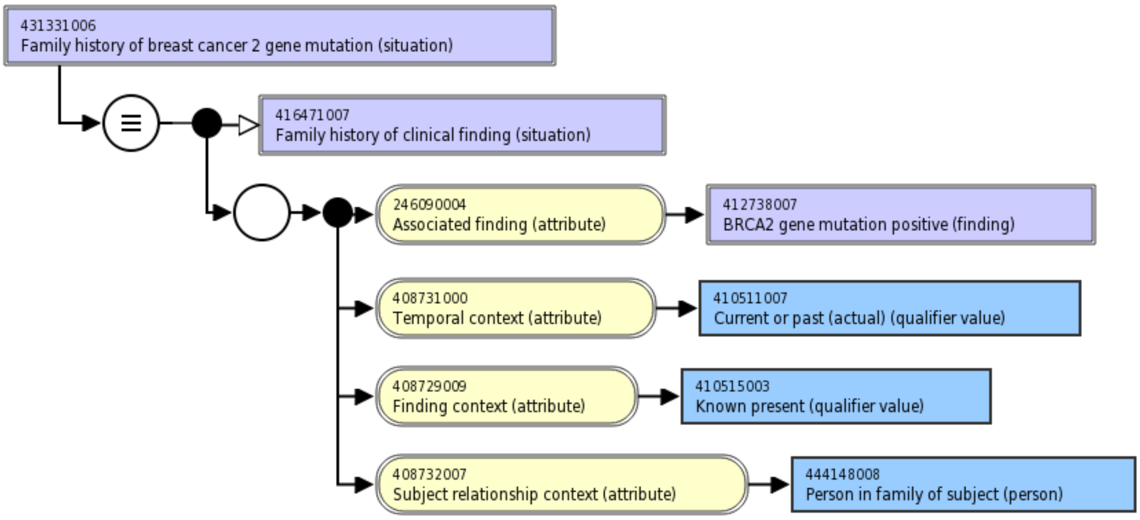

Radiology/imaging

Mammography

ICD-10

A mammography cannot be coded using ICD-10. An encounter for a screening mammography can be coded using ICD-10 CM using the following codes:

- Z12.31: Encounter for screening mammogram for malignant neoplasm of breast
- Z12.39: Encounter for other screening for malignant neoplasm of breast

An encounter for a diagnostic mammography cannot be coded using ICD-10.

SNOMED CT

A mammography can be coded with the following code:

- 71651007: Mammography (procedure), which has 19 children including:
    - 24623002: Screening mammography (procedure)

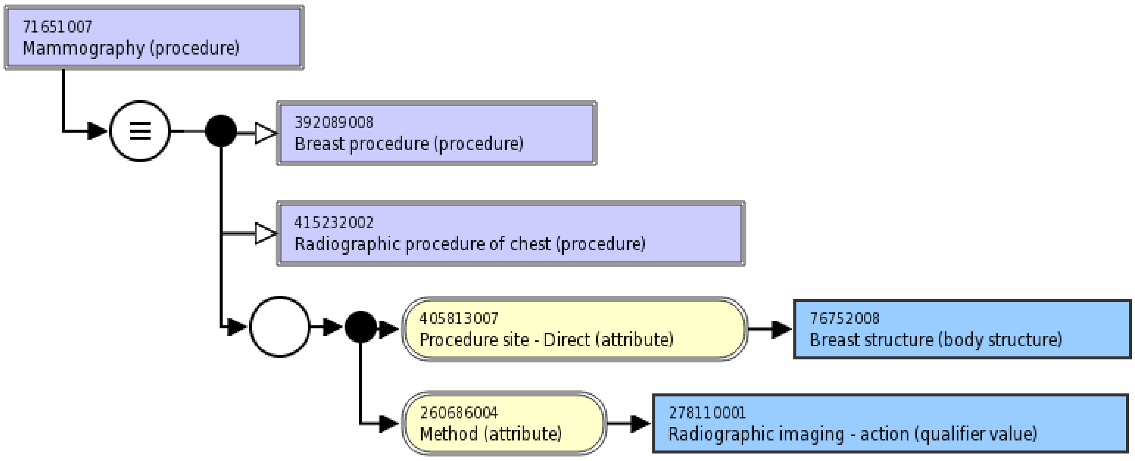

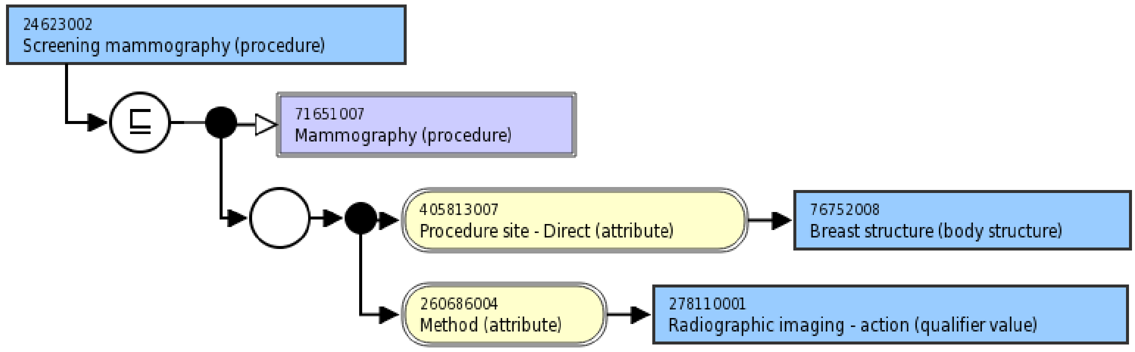

LOINC

A mammography can be coded using:

- 26175-0: Breast – bilateral mammogram screening
- 26176-8: Breast – left mammogram screening
- 26177-6: Breast – right mammogram screening
- 46356-2: Breast – unilateral mammogram screening
- 24605-8: Breast – mammogram diagnostic
- 26347-5: Breast – left mammogram diagnostic
- 26348-3: Breast – right mammogram diagnostic
- 46350-5: Breast – unilateral mammogram diagnostic
